# Prognostic Value of HALP and AHEAD Scores for Predicting 1-Month Heart Failure Following Myocardial Infarction

**DOI:** 10.3390/jcm15114363

**Published:** 2026-06-04

**Authors:** Nihat Söylemez, Burak Toprak, Özkan Karaca, Samet Yılmaz, Mehmet Ballı, Mustafa Ekici, Emrah İpek, İbrahim Halil Tanboğa

**Affiliations:** 1 Department of Cardiology, Mersin City Education and Research Hospital, Mersin 33000, Turkey; drnihatsylmz@gmail.com (N.S.); md.ozkrc@gmail.com (Ö.K.); dr_mehmetballi@hotmail.com (M.B.); 2 Department of Cardiovascular Surgery, Mersin City Education and Research Hospital, Mersin 33000, Turkey; 3 Department of Cardiology, Başkent University Adana Research Center, Adana 01120, Turkey; sametyilmaz.dr@gmail.com; 4 Department of Emergency Medicine, Mersin Provincial Health Directorate, Mersin 33000, Turkey; dr.mustafaekici@hotmail.com; 5 Department of Cardiology, İstanbul Nisantasi University Faculty of Medicine, Istanbul 34398, Turkey; emrah.ipek@nisantasi.edu.tr (E.İ.); halil.tanboga@nisantasi.edu.tr (İ.H.T.); 6 Department of Biostatistics, İstanbul Nisantasi University Faculty of Medicine, Istanbul 34398, Turkey

**Keywords:** HALP score, AHEAD score, myocardial infarction, heart failure, risk stratification, logistic regression, predictive modeling, STEMI, comorbidity index

## Abstract

**Background**: Heart failure (HF) remains a major early complication following myocardial infarction (MI), contributing significantly to morbidity and adverse clinical outcomes. Reliable early risk stratification is essential for optimizing post-MI management. This study aimed to evaluate the prognostic performance and incremental value of the HALP (Hemoglobin–Albumin–Lymphocyte–Platelet) score and the AHEAD score in predicting 1-month HF after MI. **Methods**: This retrospective cohort study included 3205 consecutive patients with MI. The primary endpoint was the development of HF within one month. Three multivariable logistic regression models were constructed: a baseline clinical model (Model 1), a HALP-integrated model (Model 2), and an AHEAD-integrated model (Model 3), with component variables excluded to avoid collinearity. Model performance was assessed using odds ratios (ORs), 95% confidence intervals (CIs), and discrimination metrics (AUC). Incremental predictive value was further evaluated using net reclassification improvement (NRI). Internal validation was performed using bootstrapping and 5-fold cross-validation. A predefined subgroup analysis was conducted in patients with preserved ejection fraction (EF ≥ 40%), excluding EF from the models. **Results**: In the full cohort, all models demonstrated high discriminative ability for 1-month HF (AUC range: 0.950–0.954), with minimal differences between models. The AHEAD-based model showed the highest point estimate (AUC = 0.954, 95% CI: 0.944–0.963), but ROC curves were largely overlapping. Despite limited changes in AUC, the AHEAD score provided moderate improvement in risk reclassification (NRI = 0.287), whereas the HALP score showed minimal incremental value (NRI = 0.152) and was not independently associated with HF in multivariable analysis. In the EF ≥ 40 subgroup, HF incidence was lower (1.9%), and model performance was attenuated but remained robust (AUC range: 0.839–0.882), with the AHEAD score retaining strong independent predictive value. Peak CKMB and creatinine were consistently associated with increased HF risk. Although the odds ratio for CKMB appeared close to unity, this reflects unit scaling, and clinically meaningful increases corresponded to substantial risk increments. A clear dose–response relationship between AHEAD score and HF probability was observed. **Conclusions**: While both HALP and AHEAD scores are associated with post-MI HF risk, only the AHEAD score provides consistent independent and incremental prognostic value beyond established clinical predictors. Its simplicity and ability to capture comorbidity burden make it a practical adjunct for early risk stratification, particularly in patients with preserved EF. However, given the minimal differences in discrimination metrics and lack of external validation, these findings should be interpreted cautiously and considered hypothesis-generating.

## 1. Introduction

Heart failure (HF) remains a major cause of morbidity and mortality following ST-elevation myocardial infarction (STEMI), despite significant advancements in reperfusion therapies such as primary percutaneous coronary intervention (PCI) [1]. The incidence of HF during the index hospitalization for acute myocardial infarction (AMI) can range from 14% to 36% [2,3]. This reality underscores a critical shift in the post-MI landscape: the clinical focus must extend beyond surviving the acute event to preserving long-term myocardial function and preventing the progression to chronic disability.

The development of HF is not a random event but reflects a pathophysiological cascade triggered by myocardial necrosis, involving adverse ventricular remodeling, neurohormonal activation, and functional decline [4]. Identifying patients at the highest risk is essential for modern post-MI care, enabling individualized strategies such as intensive surveillance, aggressive guideline-directed medical therapy (GDMT), and timely advanced interventions [2].

Traditional scores like TIMI and GRACE use baseline clinical factors to estimate mortality, but their specificity for new-onset HF is limited [5,6]. This has driven interest in more tailored tools, including nomograms and biomarker-based models [7,8]. Among these, the HALP and AHEAD scores have gained prominence.

The HALP (Hemoglobin–Albumin–Lymphocyte–Platelet) score is a dynamic immuno-nutritional biomarker reflecting inflammation and nutritional status in acute MI [9]. Low HALP scores predict in-hospital mortality and the “no-reflow” phenomenon in STEMI, highlighting acute physiological stress [10]. In contrast, the AHEAD score—atrial fibrillation, hemoglobin, elderly, abnormal renal parameters, diabetes—captures comorbidity burden [11]. By including anemia and renal dysfunction, it outperforms CHADS2 in predicting long-term mortality in CAD patients [12].

This study aims to comprehensively evaluate and compare the prognostic utility of the HALP and AHEAD scores for predicting the development of heart failure within one month following ST-elevation myocardial infarction.

## 2. Materials and Methods

### 2.1. Study Design and Population

This retrospective cohort study included 3205 consecutive patients hospitalized for ST-elevation myocardial infarction who underwent primary percutaneous coronary intervention. The primary outcome of interest was the occurrence of heart failure within one month of the index event. Heart failure was defined as either an LVEF < 40% and/or the presence of symptoms/signs of heart failure. Heart failure was defined according to contemporary ESC guidelines and required the presence of either left ventricular systolic dysfunction (LVEF < 40%) or clinical signs/symptoms of congestion requiring treatment. A pre-specified subgroup analysis was conducted on patients with a baseline left ventricular ejection fraction (EF) of 40% or greater to assess model performance in this distinct clinical population.

### 2.2. Data Collection and Variable Definitions

All patient data, including demographic, clinical, laboratory, and angiographic variables, were extracted from the provided dataset. All patients received standardized pharmacological treatment in accordance with current international STEMI management guidelines. This included dual antiplatelet therapy (aspirin plus a P2Y12 inhibitor [clopidogrel, ticagrelor, or prasugrel]), anticoagulation therapy (unfractionated heparin or low-molecular-weight heparin), high-intensity statin therapy (e.g., atorvastatin or rosuvastatin), beta-blockers (e.g., metoprolol or bisoprolol), and renin–angiotensin system inhibitors (ACE inhibitors or angiotensin receptor blockers). These treatments were initiated using standard dosing protocols and maintained consistently across the study population unless contraindicated. Therefore, inter-patient variability in pharmacological management was minimized, reducing the likelihood of treatment-related confounding in the development of early heart failure. The key predictor variables of interest were the HALP and AHEAD scores. For hemoglobin, albumin, lymphocyte, and platelet values, laboratory-specific reference intervals were used to ensure consistency in HALP and AHEAD calculations. The HALP (Hemoglobin–Albumin–Lymphocyte–Platelet) score is a continuous variable calculated from standard laboratory tests, serving as a marker of systemic inflammation and nutritional status. The AHEAD score is a simple, integer-based index (ranging from 0 to 5) reflecting a patient’s comorbidity burden. Each letter corresponds to a specific condition: atrial fibrillation, hemoglobin (anemia), elderly (age > 70), abnormal renal parameters, and diabetes mellitus.

Other covariates included in the baseline model (Model 1) were selected based on their established clinical relevance in post-MI risk stratification and included age, pain-to-door time, diastolic blood pressure (DBP), heart rate, hemoglobin, creatinine, albumin, peak creatine kinase-MB (CKMB), peak troponin, total cholesterol (TC), LDL cholesterol, triglycerides (TG), lymphocyte count, ejection fraction (EF), multi-vessel disease (MVD), and SYNTAX score.

### 2.3. Statistical Analysis

All statistical analyses were conducted using Python (version 3.12) with the pandas, numpy, scipy, statsmodels, and scikit-learn packages.

**Descriptive Statistics:** Baseline patient characteristics were stratified by the 1-month heart failure outcome. Continuous variables were summarized using means and standard deviations (SDs) and were compared between groups with a Student’s *t*-test for independent samples. Categorical variables were summarized as frequencies and percentages and compared using the chi-square test. A two-sided *p*-value < 0.05 was considered statistically significant for all tests.

**Multivariable Logistic Regression Modeling:** Three multivariable logistic regression models were developed to predict the binary outcome of 1-month heart failure in the full cohort: Model 1 (Baseline Clinical Model) included age, pain-to-door time, DBP, heart rate, hemoglobin, creatinine, albumin, peak CKMB, peak troponin, TC, LDL, TG, EF, MVD, syntax score, and lymphocyte count. Model 2 (HALP-Integrated Model) included the variables from Model 1, but hemoglobin, lymphocyte count, and albumin were replaced by the composite HALP score. Model 3 (AHEAD-Integrated Model) included the same variables as Model 1, except that age, hemoglobin, and creatinine—all components of the AHEAD score—were removed to avoid collinearity.

The same set of three models was subsequently developed and tested in the subgroup of patients with EF ≥ 40, with the EF variable excluded from all models in this subgroup analysis. Models were fitted using the generalized linear model function from the statsmodels library with a binomial family and logit link function. All continuous variables were checked for multicollinearity, and variance inflation factors (VIFs) were below 2.0 for all predictors. Results were reported as odds ratios (ORs) with their corresponding 95% confidence intervals (CIs) and *p*-values. Odds ratios for continuous variables are expressed per one-unit increase in the original measurement scale unless otherwise specified. For variables with large numerical ranges (e.g., peak CKMB, pain-to-door time), even small per-unit increases may correspond to clinically meaningful changes when considered over larger increments.

**Model Performance Evaluation:** Model performance was evaluated on two key domains: discrimination and calibration. **Discrimination:** The ability of each model to distinguish between patients who did and did not develop heart failure was assessed using the area under the receiver operating characteristic curve (AUC). 95% CIs for the AUC values were calculated using a non-parametric bootstrapping method with 1000 resamples to provide a robust measure of uncertainty. In addition, to further assess model stability and reduce the risk of overfitting, internal validation was performed using 5-fold cross-validation. The dataset was randomly partitioned into five subsets, and model training and evaluation were iteratively performed across folds. Mean AUC values obtained from cross-validation were used to evaluate the robustness and generalizability of the models within the available dataset. ROC curves for the three models were overlaid on a single plot for direct visual comparison. Calibration: Calibration refers to the agreement between predicted probabilities and observed outcomes. It was assessed visually using calibration plots, which graph the observed event rate against the predicted probability for deciles of risk. A perfectly calibrated model would have points falling on the 45-degree diagonal line.

**Visualizations:** Partial effect plots were generated for the HALP, AHEAD, and EF variables to visualize their independent, marginal effects on the predicted probability of heart failure while holding all other model covariates at their mean values. Additionally, histograms were created to display the distributions of HALP and AHEAD scores for the entire population, the no-HF group, and the HF group, with overlays indicating the mean, SD, median, and interquartile range.

## 3. Results

**Baseline Patient Characteristics**

The full cohort consisted of 3205 patients. Among them, 356 (11.1%) developed heart failure within one month. The baseline characteristics of patients with and without heart failure are presented in Table 1. Patients who developed HF were significantly older and had longer pain-to-door times, lower DBP, and higher heart rates. HF patients also had significantly lower hemoglobin and albumin levels, higher creatinine, and markedly elevated cardiac enzymes (peak CKMB and troponin). Furthermore, HF patients had significantly lower ejection fractions and higher SYNTAX scores, indicating greater severity of cardiac dysfunction and coronary artery disease (Table 1).

**Full Cohort Logistic Regression Models**

The results of the three multivariable logistic regression models for the full cohort are summarized in Table 2.

**Model 1 (Baseline):** In the baseline model, higher LVEF (OR = 0.737, *p* < 0.001) was strongly protective against HF. Conversely, higher age, longer pain-to-door time, lower DBP, lower hemoglobin, higher creatinine, lower albumin, higher peak CKMB, and higher TG were all significant independent predictors of 1-month HF (Table 2).

**Model 2 (HALP):** After replacing its constituent components with the HALP score, the model showed that higher age, longer pain-to-door time, lower DBP, higher creatinine, higher peak CKMB, higher TG, and lower EF remained significant predictors (Table 2). The HALP score itself was not statistically significant (OR = 0.911, *p* = 0.785) in this multivariable context, indicating a lack of independent predictive value after adjustment for its constituent variables.

**Model 3 (AHEAD):** When the AHEAD score was included, it emerged as a powerful and highly significant predictor of heart failure (OR = 1.786, 95% CI: 1.522–2.097, *p* < 0.001). Other significant predictors in this model included longer pain-to-door time, higher peak CKMB, and lower EF (Table 2).

**Model Performance in the Full Cohort**

All three models demonstrated excellent discrimination in predicting 1-month HF [Model 1 AUC: 0.952 (95% CI: 0.941–0.961), Model 2 AUC: 0.950 (95% CI: 0.939–0.960), Model 3 AUC: 0.954 (95% CI: 0.944–0.963)].

**Figure 1:**
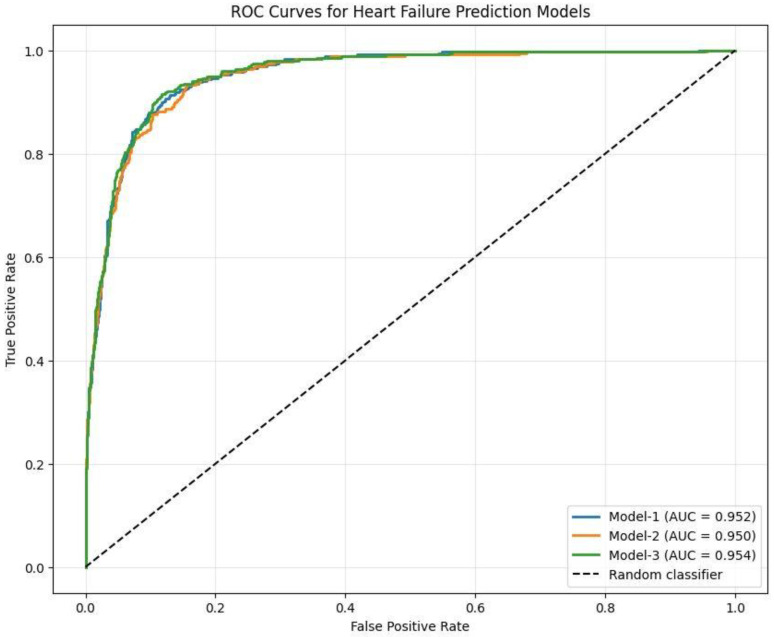
Receiver operating characteristic (ROC) curves for the three logistic regression models predicting 1-month heart failure in the full patient cohort (*n* = 3205). Model 1 is the baseline clinical model, Model 2 incorporates the HALP score, and Model 3 incorporates the AHEAD score. The area under the curve (AUC) for each model is shown. The dashed line represents a random classifier (AUC = 0.5).

The ROC curves for the three models were nearly superimposable, as shown in Figure 1, indicating comparable and excellent discriminative ability (Figure 1).

To further evaluate the incremental predictive value of the HALP and AHEAD scores beyond the baseline clinical model, net reclassification improvement (NRI) analysis was performed. The addition of the HALP score resulted in a limited improvement in risk classification (NRI = 0.152), consistent with its lack of independent significance in multivariable analysis. In contrast, the AHEAD-based model demonstrated a moderate improvement in patient reclassification (NRI = 0.287), despite only minimal differences in AUC. These findings suggest that while changes in discrimination metrics were small, the AHEAD score contributed meaningfully to risk stratification at the individual patient level. The calibration plots (Supplemental Figure S1) revealed good agreement between predicted and observed probabilities for all models, with points lying close to the ideal diagonal line.

Internal validation using 5-fold cross-validation demonstrated consistent model performance across all three models. The cross-validated AUC values were slightly lower than the original estimates (Model 1: 0.938, Model 2: 0.934, Model 3: 0.941), indicating minimal optimism bias and supporting the robustness of the models (Table 3).

**Partial Effect Plots and Histograms**

The partial effect plots (Supplemental Figure S2) illustrated the marginal impact of key variables. There was a steep, inverse relationship between EF and HF risk. Conversely, the probability of HF increased sharply with a rising AHEAD score. The relationship with the HALP score was less pronounced and relatively flat, consistent with its non-significance in the multivariable model.

Histograms for HALP and AHEAD (Supplemental Figure S3) showed distinct distributions between the HF and no-HF groups. Patients who developed HF had visibly lower HALP scores and higher AHEAD scores compared to those who did not, with distributions shifted to the left and right, respectively.

**Subgroup Analysis: Patients with EF ≥ 40**

A total of 2562 patients (79.9% of the cohort) had an EF of 40% or greater. Within this subgroup, only 49 patients (1.9%) developed HF.

The results of the logistic regression models for this subgroup are shown in Table 4. Model 1 (EF ≥ 40): Significant predictors included longer pain-to-door time, lower DBP, higher creatinine, lower albumin, and higher peak CKMB. Model 2 (EF ≥ 40): Similar predictors were significant, with higher creatinine showing a stronger effect (OR = 1.763, *p* < 0.001). HALP was not significant. Model 3 (EF ≥ 40): The AHEAD score remained a very strong and highly significant predictor (OR = 2.060, 95% CI: 1.536–2.762, *p* < 0.001). Model performance in this subgroup was attenuated compared to the full cohort but remained strong [Model 1 (EF ≥ 40) AUC: 0.871 (95% CI: 0.827–0.911), Model 2 (EF ≥ 40) AUC: 0.839 (95% CI: 0.779–0.891), and Model 3 (EF ≥ 40) AUC: 0.882 (95% CI: 0.839–0.919)] (Table 4).

**Figure 2:**
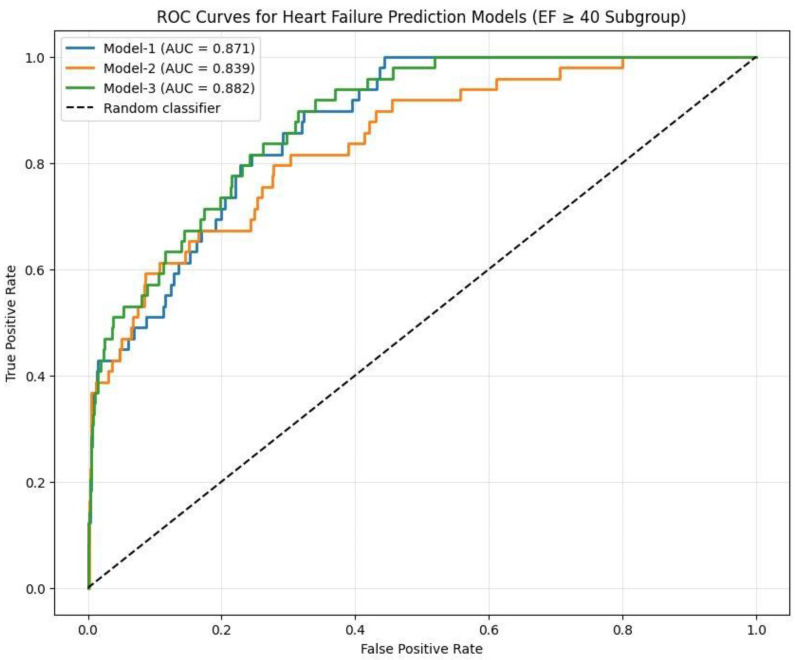
ROC curves for the three logistic regression models predicting 1-month heart failure in the subgroup of patients with ejection fraction ≥ 40 (*n* = 2562). EF was not included as a predictor in these models.

The ROC curves (Figure 2) and calibration plots (Supplemental Figure S4) for the subgroup confirmed the good performance of the models, with the AHEAD-based model showing the best discrimination.

Partial effect plots (Supplemental Figure S5) and histograms (Supplemental Figure S6) within this subgroup reinforced the strong positive association between the AHEAD score and HF risk.

## 4. Discussion

This study provides a comprehensive evaluation of two novel scoring systems, HALP and AHEAD, for the prediction of 1-month heart failure following myocardial infarction. Our analysis demonstrates that while established clinical and laboratory markers provide a strong baseline for prediction, incorporating a simple comorbidity index like the AHEAD score can further enhance risk stratification, particularly in patient subgroups where traditional markers like EF are less informative.

Our primary finding is the excellent predictive performance of all three logistic regression models in the full patient cohort, with AUCs exceeding 0.95. Such high levels of discrimination are uncommon in clinical prediction models and should be interpreted with caution. Several factors may have contributed to the observed performance. First, the inclusion of strong predictors closely linked to the outcome, particularly ejection fraction and peak CKMB, likely enhanced model discrimination. Second, the definition of heart failure, which incorporates reduced LVEF, introduces a degree of predictor–outcome overlap that may have artificially inflated AUC values. Third, the use of a single retrospective dataset raises the possibility of model overfitting, despite the application of internal validation techniques. In addition, established biomarkers reflecting myocardial stress and overload, particularly NT-proBNP, were not consistently available in the study dataset and therefore could not be incorporated into the predictive models or comparative analyses. Since NT-proBNP is a well-established biomarker for heart failure diagnosis and prognosis, its absence represents an important limitation, and future studies integrating both biomarker-based and clinical risk models may provide more comprehensive prognostic assessment. Taken together, these considerations suggest that the observed high AUC values may partially reflect dataset-specific characteristics rather than purely generalizable predictive performance. However, the definition of heart failure, which includes reduced left ventricular ejection fraction (LVEF < 40%), may partially overlap with predictors used in the models, particularly EF itself. This overlap may have contributed to the high observed discrimination performance and should be interpreted with caution. To address this potential limitation, a predefined subgroup analysis was conducted in patients with preserved ejection fraction (EF ≥ 40%), in which EF was excluded from the predictive models. In this subgroup, although overall model performance was attenuated, the models retained good discriminative ability, and the AHEAD score remained a significant predictor. Furthermore, the use of NRI analysis demonstrated that, despite minimal changes in AUC, the AHEAD score provided additional value in patient-level risk reclassification. These findings support the robustness of the results while highlighting that the incremental benefit of these models should be interpreted within the context of potential predictor–outcome overlap. This indicates that the combination of demographic, clinical, laboratory, and angiographic variables robustly identifies patients at high risk for early HF. Notably, Model 3, which incorporated the AHEAD score, achieved the highest point estimate for AUC (0.954). However, the absolute differences between models were minimal, and the ROC curves were nearly overlapping, indicating that this apparent superiority should be interpreted cautiously. The AHEAD score itself was a powerful independent predictor, with each one-point increase conferring a 79% higher odds of developing HF.

The analysis of the HALP score yielded different results. While patients who developed HF had significantly lower mean HALP scores in the univariate analysis, the HALP score was not a significant independent predictor in the multivariable model (Model 2). This suggests that most of its prognostic contribution is already incorporated through its individual biochemical components, which remain strongly represented in the baseline model (hemoglobin, albumin, etc.) and other clinical variables already included in the baseline model, such as creatinine and peak CKMB, which also reflect systemic stress and inflammation. Furthermore, when the incremental predictive performance of the models was formally assessed using net reclassification improvement (NRI), a divergence between discrimination and reclassification metrics became apparent. While differences in AUC between models were minimal, the AHEAD score demonstrated a moderate improvement in patient-level risk reclassification (NRI = 0.287), suggesting a clinically meaningful contribution beyond traditional predictors. In contrast, the HALP score provided only limited reclassification benefit (NRI = 0.152), consistent with its lack of independent significance in multivariable analysis. These findings indicate that although the incremental value of these composite scores may appear modest when evaluated solely by AUC, the AHEAD score in particular may still enhance individualized risk stratification.

The subgroup analysis of patients with EF ≥ 40 is a key contribution of this study. This group, representing nearly 80% of the cohort, has a much lower absolute risk of HF (1.9%). As expected, the predictive performance of the models was attenuated in this lower-risk population. However, discrimination remained strong, with AUCs ranging from 0.839 to 0.882. Crucially, the AHEAD score maintained its strong, independent predictive power in this subgroup (OR = 2.060, *p* < 0.001), and the AHEAD-based model once again demonstrated the best performance (AUC = 0.882). This finding is clinically significant, as it highlights the AHEAD score’s ability to stratify risk even when the most powerful single predictor, a severely reduced EF, is absent. Nevertheless, the distinction between statistical and clinical significance should be carefully considered. Although the AHEAD-based model demonstrated a numerically higher AUC, the ROC curves were largely overlapping, indicating minimal improvement in overall discrimination. This suggests that the added predictive value of the AHEAD score beyond strong established predictors such as ejection fraction and peak CKMB may be limited when assessed solely by conventional discrimination metrics. However, NRI analysis revealed a moderate improvement in patient-level risk reclassification (NRI = 0.287), indicating that the AHEAD score may still provide clinically relevant refinement in individual risk assessment despite minimal changes in AUC. Therefore, its role may be better interpreted as complementary rather than substitutive within existing risk models. From a practical perspective, the clinical utility of the AHEAD score lies in its simplicity, immediate availability, and ease of bedside application without requiring additional laboratory assays or advanced imaging parameters. Unlike biomarkers such as NT-proBNP, which may not be routinely available in all healthcare settings and may increase healthcare costs, the AHEAD score is derived entirely from commonly accessible clinical variables. Therefore, rather than replacing established biomarkers or conventional risk models, the AHEAD score may serve as a rapid adjunctive tool for early identification of patients who may benefit from closer follow-up, intensified guideline-directed medical therapy, or more careful surveillance during the early post-myocardial infarction period. From a clinical standpoint, the integration of the AHEAD score into early post-MI risk assessment may provide actionable guidance for therapeutic intensification. Importantly, the translation of risk stratification into clinical benefit depends on the timely initiation and optimization of guideline-directed medical therapy (GDMT). In this context, patients identified as high-risk based on elevated AHEAD scores may represent an ideal target population for early and intensified therapeutic strategies. Contemporary evidence supports a more proactive approach to post-MI management, including early initiation of disease-modifying therapies. For instance, simplified treatment strategies such as the cardiovascular polypill have been proposed to improve adherence and facilitate rapid implementation of secondary prevention measures [13]. In addition, sodium–glucose cotransporter-2 (SGLT2) inhibitors, such as empagliflozin and dapagliflozin, have demonstrated significant reductions in cardiovascular mortality and sudden cardiac death, reinforcing their role as early cornerstone therapies in high-risk patients [14]. Furthermore, novel agents targeting the nitric oxide-soluble guanylate cyclase pathway, such as vericiguat, have shown benefit in patients with worsening heart failure and may represent an additional therapeutic option in selected high-risk individuals [15].

Taken together, these findings highlight that the clinical utility of the AHEAD score lies not only in risk prediction but also in its potential to guide early, tailored, and intensified therapeutic strategies. Therefore, integrating risk stratification tools with evidence-based treatment pathways may help bridge the gap between prognostic assessment and improved clinical outcomes. In our cohort, a higher AHEAD score was consistently associated with a markedly elevated risk of developing heart failure within the first month, suggesting that this comorbidity-driven index functions not only as a prognostic marker but also as an anticipatory signal for clinical deterioration. Accordingly, patients presenting with elevated AHEAD scores may benefit from a more proactive management approach, including lower thresholds for intensive care monitoring, closer hemodynamic and congestion surveillance, and early initiation or accelerated uptitration of guideline-directed medical therapy. Moreover, because the components of the AHEAD score—advanced age, anemia, renal dysfunction, diabetes mellitus, and atrial fibrillation—represent modifiable or optimizable contributors to HF progression, targeted interventions such as renal function optimization, correction of hemoglobin deficits, strict glycemic control, and rhythm or rate management may attenuate subsequent HF development. Thus, incorporating the AHEAD score into routine clinical workflows has the potential to transform post-MI management from a reactive to a preventive strategy, particularly in patients with preserved ejection fraction, in whom traditional risk markers may be less discriminatory.

Our findings align with and extend existing literature on post-MI risk stratification. Among established biomarkers, N-terminal pro-B-type natriuretic peptide (NT-proBNP) has consistently demonstrated strong prognostic value in cardiovascular disease. Elevated NT-proBNP levels reflect myocardial wall stress, ventricular dysfunction, and neurohormonal activation and have been associated with adverse cardiovascular outcomes, including early heart failure and cardiogenic shock. Duchnowski and Śmigielski demonstrated the usefulness of myocardial damage biomarkers, including NT-proBNP, in predicting cardiogenic shock in patients undergoing heart valve surgery, further supporting the clinical relevance of biomarkers reflecting myocardial overload and injury [16]. In this context, the prognostic performance observed with the AHEAD score in our study should be interpreted within the broader framework of established biomarkers associated with myocardial stress and adverse cardiac remodeling. The importance of age, renal function, cardiac enzyme levels, and EF are well-established predictors, consistent with models like the GRACE and TIMI scores [5,6,17]. Our baseline model (Model 1) confirms the utility of this multimodal approach. Importantly, all patients were treated with a uniform guideline-based pharmacological strategy, which minimizes treatment-related heterogeneity and strengthens the internal validity of the observed associations. However, it should be acknowledged that the present models were primarily constructed using readily available clinical and laboratory variables at admission and did not incorporate detailed procedural characteristics of the index intervention. While this approach enhances clinical applicability and early risk stratification, it may not fully capture the impact of procedural complexity and reperfusion quality on subsequent heart failure development.

The strong performance of the AHEAD score is consistent with studies that have validated it as a powerful predictor of long-term mortality in patients with acute and chronic coronary syndromes [12,18]. Our study is novel in its application of AHEAD specifically to the endpoint of early post-MI heart failure. The score’s strength lies in its pragmatic composition; it integrates five conditions (atrial fibrillation, anemia, advanced age, abnormal renal function, and diabetes) that are known to synergistically worsen cardiovascular outcomes through mechanisms such as neurohormonal activation, volume overload, and systemic inflammation [11]. Its superiority over CHADS2 in CAD populations, as noted in previous literature, is attributable to its inclusion of anemia and renal dysfunction [12], which our baseline analysis also identified as significant HF predictors.

The non-significance of the HALP score in our multivariable model contrasts with some studies in STEMI patients where it independently predicted in-hospital mortality and “no-reflow” [10]. This discrepancy may be due to differences in study endpoints (mortality vs. HF), cohort composition, and the variables included in the multivariable adjustment. Several studies have shown that HALP is more strongly associated with short-term inflammatory responses and mortality rather than subacute heart failure development, which may explain its limited independent significance in our study. As a dynamic marker, HALP may be more predictive of very acute events and severe inflammatory responses than the subacute development of HF over one month, where underlying comorbidities and the extent of myocardial damage (captured by CKMB and EF) may play a more dominant role.

The results of this study have several practical implications. First, the AHEAD score is a simple, easy-to-calculate tool that uses readily available data at admission. Its robust performance suggests it could be integrated into initial risk assessment to identify patients who, regardless of their initial EF, carry a high burden of comorbidity and are thus predisposed to developing HF. A high AHEAD score could trigger more intensive inpatient monitoring, more aggressive GDMT, and a lower threshold for specialist consultation.

Second, the strong performance of the models in the EF ≥ 40 subgroup underscores the concept that post-MI HF is not solely a problem of reduced systolic function. In patients with preserved or mildly reduced EF, the cumulative impact of comorbidities, as quantified by the AHEAD score, is a primary driver of risk. This supports a more holistic approach to post-MI care that moves beyond an exclusive focus on LVEF. From a clinical perspective, the practical value of the AHEAD score lies not in replacing established predictors but in complementing them by incorporating comorbidity burden into early risk assessment. Its ease of use and availability at admission may facilitate rapid bedside stratification, particularly in settings where comprehensive imaging or advanced biomarkers are not immediately accessible.

This study’s strengths include its large cohort size, the direct comparison of a dynamic immuno-nutritional marker (HALP) with a static comorbidity index (AHEAD), and the robust subgroup analysis in a clinically important population (EF ≥ 40).

However, several limitations must be acknowledged. The analysis is retrospective, which introduces the potential for selection bias and unmeasured confounding. As a retrospective observational analysis, this study is inherently subject to several methodological limitations. First, the retrospective design may introduce selection bias, as patient inclusion was dependent on the availability and completeness of recorded data rather than predefined prospective criteria. Second, residual confounding cannot be fully excluded despite multivariable adjustment, particularly for variables that were not captured or systematically recorded, such as detailed hemodynamic parameters, treatment variations, or temporal changes in clinical management. Third, causal relationships cannot be established due to the non-randomized nature of the study, and the observed associations should therefore be interpreted as hypothesis-generating rather than definitive evidence. The dataset is from a single source, which may limit the generalizability of our findings to other populations. Another important limitation of this study is the lack of detailed procedural and acute-phase interventional data. Variables such as door-to-balloon time, contrast volume, number of treated lesions, type of revascularization strategy, and device-specific characteristics (e.g., stent or drug-coated balloon type) were not consistently available in the dataset. These factors are known to influence myocardial injury extent, reperfusion success, and subsequent ventricular remodeling and therefore may play a significant role in the early development of heart failure. The absence of these procedural parameters may have limited the comprehensiveness of the predictive models and could have introduced residual confounding. Future studies incorporating detailed procedural and peri-interventional variables are warranted to provide a more refined and clinically integrated risk stratification. The definitions of variables, including heart failure, were based on the available data and could not be independently adjudicated. Finally, while our models showed excellent discrimination, prospective validation is required before they can be recommended for widespread clinical use. Another important limitation of the present study is the lack of prospective validation in an independent cohort. Although the predictive models demonstrated excellent discrimination within the current dataset, their performance was evaluated using internal data only. As a result, the potential for overfitting cannot be entirely excluded, and the generalizability of the findings to different populations or clinical settings remains uncertain. However, the application of both bootstrapping and cross-validation techniques provides additional reassurance regarding the internal stability of the models. The close agreement between original and cross-validated AUC values suggests that overfitting is unlikely to fully account for the observed high predictive performance. External validation in a prospective, independent cohort would be essential to confirm the robustness, reproducibility, and real-world applicability of the proposed models. In the absence of such validation, the generalizability of the findings remains uncertain, and model performance may differ across populations with varying clinical characteristics and treatment patterns. Therefore, the current findings should be interpreted with caution and considered as hypothesis-generating until further validated.

Future research should focus on prospectively validating the AHEAD score for HF prediction in diverse, multi-ethnic post-MI cohorts. Investigating the potential for combining dynamic markers like HALP (or its components) with static scores like AHEAD to create a more powerful, integrated risk model could be a fruitful avenue. Furthermore, linking high-risk designations from these models to specific interventions and demonstrating improved patient outcomes would be the ultimate validation of their clinical utility.

## 5. Conclusions

In conclusion, this analysis confirms that a multivariable approach is highly effective for predicting 1-month heart failure after MI. The AHEAD score, a simple index of comorbidity burden, is a potent and independent predictor of this outcome, both in the overall population and in the important subgroup of patients with preserved EF. Its ease of use and consistent performance suggest that it may serve as a supportive tool for risk stratification in post-MI patient care, although its incremental clinical benefit beyond established predictors appears modest and requires further validation. Furthermore, the AHEAD score may offer supportive clinical utility by identifying patients who could benefit from closer monitoring and tailored management strategies; however, its incremental benefit beyond established predictors and potential overlap with outcome definitions should be interpreted with caution.

## Figures and Tables

**Table 1:** Baseline characteristics of patients with and without 1-month heart failure (*n* = 3205).

Variables	Heart Failure = No (*n* = 2849)	Heart Failure = Yes (*n* = 356)	*p*-Value
Age, years	58.11 ± 11.72	62.83 ± 12.50	**<0.001**
Sex (% Male)	75.6%	64.0%	**<0.001**
Diabetes mellitus (% yes)	21.2%	41.3%	**<0.001**
Hypertension (% yes)	42.5%	46.1%	0.226
Smoking (% yes)	55.6%	39.6%	**<0.001**
Hyperlipidemia (% yes)	41.0%	36.2%	0.098
Family history of CAD (% yes)	22.3%	16.6%	**0.016**
Previous MI (% yes)	11.4%	15.4%	**0.031**
Previous revascularization (% yes)	11.9%	12.6%	0.749
Previous antiplatelet (% yes)	13.7%	16.9%	0.119
Previous betablocker (% yes)	12.0%	11.8%	0.994
Previous statin (% yes)	19.9%	16.6%	0.155
Previous ACE (% yes)	19.8%	22.2%	0.328
MI pattern (% anterior)	44.6%	76.1%	**<0.001**
KILLIP (Class > I)	13.0%	44.9%	**<0.001**
Multivessel disease (% > I vessel)	40.3%	55.6%	**<0.001**
TIMI flow (Grade < III)	99.5%	96.9%	**<0.001**
Atrial fibrillation (% yes)	3.5%	19.4%	**<0.001**
Pain-to-door time (min)	170.07 ± 108.69	240.24 ± 132.11	**<0.001**
SBP, mmHg	133 ± 27.3	128 ± 46.5	0.067
DBP, mmHg	77.88 ± 16.59	73.18 ± 28.19	**<0.001**
Heart rate, beat/min	76.72 ± 14.94	82.06 ± 24.29	**<0.001**
Hemoglobin, g/dL	13.63 ± 1.85	12.98 ± 2.39	**<0.001**
Creatinine, mg/dL	0.93 ± 0.42	1.17 ± 0.65	**<0.001**
Albumin, mg/dL	3.76 ± 0.47	3.51 ± 0.59	**<0.001**
Peak CKMB	195.41 ± 131.67	439.66 ± 229.08	**<0.001**
Peak troponin	94.24 ± 81.49	216.78 ± 116.89	**<0.001**
Total cholesterol, mg/dL	178.88 ± 40.34	170.92 ± 42.08	**0.048**
LDL cholesterol, mg/dL	114.42 ± 35.47	108.15 ± 36.27	**0.017**
Triglycerides, mg/dL	139.81 ± 86.17	128.63 ± 60.75	0.176
LVEF (%)	47.99 ± 6.86	34.24 ± 5.73	**<0.001**
Syntax score	15.09 ± 6.98	18.63 ± 8.23	**<0.001**
Lymphocyte count	2.00 ± 1.00	1.80 ± 1.13	**0.044**
HALP	0.42 ± 0.26	0.34 ± 0.28	**<0.001**
AHEAD	0.68 ± 0.87	1.55 ± 1.32	**<0.001**

Statistical tests applied include Student’s *t*-test for continuous variables and the chi-square test for categorical variables. Significant values are indicated in bold. The *p*-value reflects the level of statistical significance, with values <0.05 considered statistically significant. CAD = coronary artery disease; MI = myocardial infarction; TIMI = thrombolysis in myocardial infarction; SBP = systolic blood pressure; DBP = diastolic blood pressure; CKMB = creatine kinase-MB; LDL = low-density lipoprotein; LVEF = left ventricular ejection fraction.

**Table 2:** Multivariable logistic regression models for predicting 1-month heart failure (full cohort) (*n* = 3205).

Model	Variable	Odds Ratio	95% CI Lower	95% CI Upper	*p*-Value
**Model-1**					
	Age	1.021	1.006	1.038	**0.007**
	Pain-to-door time	1.002	1.000	1.003	**0.017**
	DBP	0.992	0.985	1.000	**0.045**
	Hemoglobin	0.878	0.809	0.953	**0.002**
	Creatinine	1.644	1.265	2.136	**<0.001**
	Albumin	0.677	0.475	0.965	**0.031**
	Peak CKMB	1.002	1.001	1.004	**<0.001**
	Triglycerides	1.003	1.000	1.005	**0.047**
	LVEF	0.737	0.710	0.765	**<0.001**
**Model-2**					
	Age	1.033	1.018	1.048	**<0.001**
	Pain-to-door time	1.002	1.000	1.003	**0.007**
	DBP	0.991	0.983	0.998	**0.011**
	Creatinine	1.783	1.379	2.306	**<0.001**
	Peak CKMB	1.002	1.001	1.003	**<0.001**
	Triglycerides	1.003	1.000	1.006	**0.029**
	LVEF	0.737	0.710	0.765	**<0.001**
	HALP	0.911	0.466	1.780	0.785
**Model-3**					
	Pain-to-door time	1.001	1.000	1.003	**0.045**
	Peak CKMB	1.002	1.001	1.003	**<0.001**
	LVEF	0.740	0.713	0.769	**<0.001**
	AHEAD	1.786	1.522	2.097	**<0.001**

Statistical tests applied include multivariable logistic regression analyses. Significant values are indicated in bold. The *p*-value reflects the level of statistical significance, with values < 0.05 considered statistically significant. Odds ratios for continuous variables represent the change in risk associated with a one-unit increase in the respective variable. CI = confidence interval; DBP = diastolic blood pressure; CKMB = creatine kinase-MB; LVEF = left ventricular ejection fraction; HALP = Hemoglobin–Albumin–Lymphocyte–Platelet score; AHEAD = Atrial fibrillation–Hemoglobin–Elderly–Abnormal renal function–Diabetes score.

**Table 3:** Internal validation with cross-validation.

Model	Original AUC	Cross-Validated AUC	Interpretation
Model 1	0.952	**0.938**	Stable performance with minimal optimism
Model 2	0.95	**0.934**	Consistent but no incremental improvement
Model 3	0.954	**0.941**	Robust with minimal optimism

Internal validation of model performance was conducted using 5-fold cross-validation to assess robustness and potential overfitting. The dataset was randomly partitioned into five equal subsets, and models were iteratively trained and tested across folds. Cross-validated AUC values represent the mean performance across all folds and provide an estimate of model generalizability within the available dataset. The comparison between original and cross-validated AUC values reflects the degree of optimism bias, with minimal differences indicating stable and reliable model performance. HALP = Hemoglobin–Albumin–Lymphocyte–Platelet score; AHEAD = Atrial fibrillation–Hemoglobin–Elderly–Abnormal renal function–Diabetes score; AUC = area under the curve.

**Table 4:** Multivariable logistic regression models for predicting 1-month heart failure (EF ≥ 40 subgroup) (*n* = 2562).

Model	Variable	Odds Ratio	95% CI Lower	95% CI Upper	*p*-Value
**Model-1 (EF ≥ 40)**					
	Pain-to-door time	1.004	1.001	1.006	**0.003**
	DBP	0.977	0.961	0.993	**0.006**
	Creatinine	1.587	1.151	2.187	**0.005**
	Albumin	0.391	0.168	0.914	**0.030**
	Peak CKMB	1.004	1.001	1.007	**0.003**
**Model-2 (EF ≥ 40)**					
	Age	1.030	1.001	1.060	**0.041**
	Pain-to-door time	1.004	1.001	1.006	**0.003**
	DBP	0.975	0.959	0.991	**0.002**
	Creatinine	1.763	1.306	2.379	**<0.001**
	Peak CKMB	1.004	1.001	1.007	**0.004**
	Peak troponin	1.004	1.000	1.008	**0.039**
	Triglycerides	1.005	1.001	1.010	**0.026**
**Model-3 (EF ≥ 40)**					
	Pain-to-door time	1.004	1.001	1.006	**0.002**
	DBP	0.983	0.968	0.999	**0.036**
	Peak CKMB	1.004	1.001	1.006	**0.007**
	AHEAD	2.060	1.536	2.762	**<0.001**

Statistical tests applied include multivariable logistic regression analyses. Significant values are indicated in bold. The *p*-value reflects the level of statistical significance, with values < 0.05 considered statistically significant. Odds ratios for continuous variables represent the change in risk associated with a one-unit increase in the respective variable. CI = confidence interval; DBP = diastolic blood pressure; CKMB = creatine kinase-MB; HALP = Hemoglobin–Albumin–Lymphocyte–Platelet score; AHEAD = Atrial fibrillation–Hemoglobin–Elderly–Abnormal renal function–Diabetes score.

## Data Availability

The data that support the findings of this study are available from the corresponding author upon reasonable request.

## Supplementary Materials

The following supporting information can be downloaded at: https://www.mdpi.com/article/10.3390/jcm15114363/s1, Figure S1: Calibration plots for the three logistic regression models in the full cohort; Figure S2: Partial effect plots of EF, AHEAD, and HALP scores; Figure S3: Distribution histograms of HALP and AHEAD scores; Figure S4: Calibration plots in the EF ≥ 40 subgroup; Figure S5: Partial effect plots in the EF ≥ 40 subgroup; Figure S6: Distribution histograms in the EF ≥ 40 subgroup.

## Abbreviations

AUC	Area under the curve
CKMB	Creatine kinase-MB
CI	Confidence interval
DBP	Diastolic blood pressure
EF	Ejection fraction
GDMT	Guideline-directed medical therapy
HALP	Hemoglobin–Albumin–Lymphocyte–Platelet score
HF	Heart failure
LDL	Low-density lipoprotein
LVEF	Left ventricular ejection fraction
MI	Myocardial infarction
MVD	Multivessel disease
OR	Odds ratio
PCI	Percutaneous coronary intervention
ROC	Receiver operating characteristic
SBP	Systolic blood pressure
STEMI	ST-elevation myocardial infarction
SYNTAX score	Coronary Artery Disease Complexity Score
TG	Triglycerides
