# Supplementary figures and images for: Prognostic Value of HALP and AHEAD Scores for Predicting 1-Month Heart Failure Following Myocardial Infarction
Source: J Clin Med. 2026 Jun 4;15(11):4363. doi: 10.3390/jcm15114363 (PMC13257798; doi:10.3390/jcm15114363)

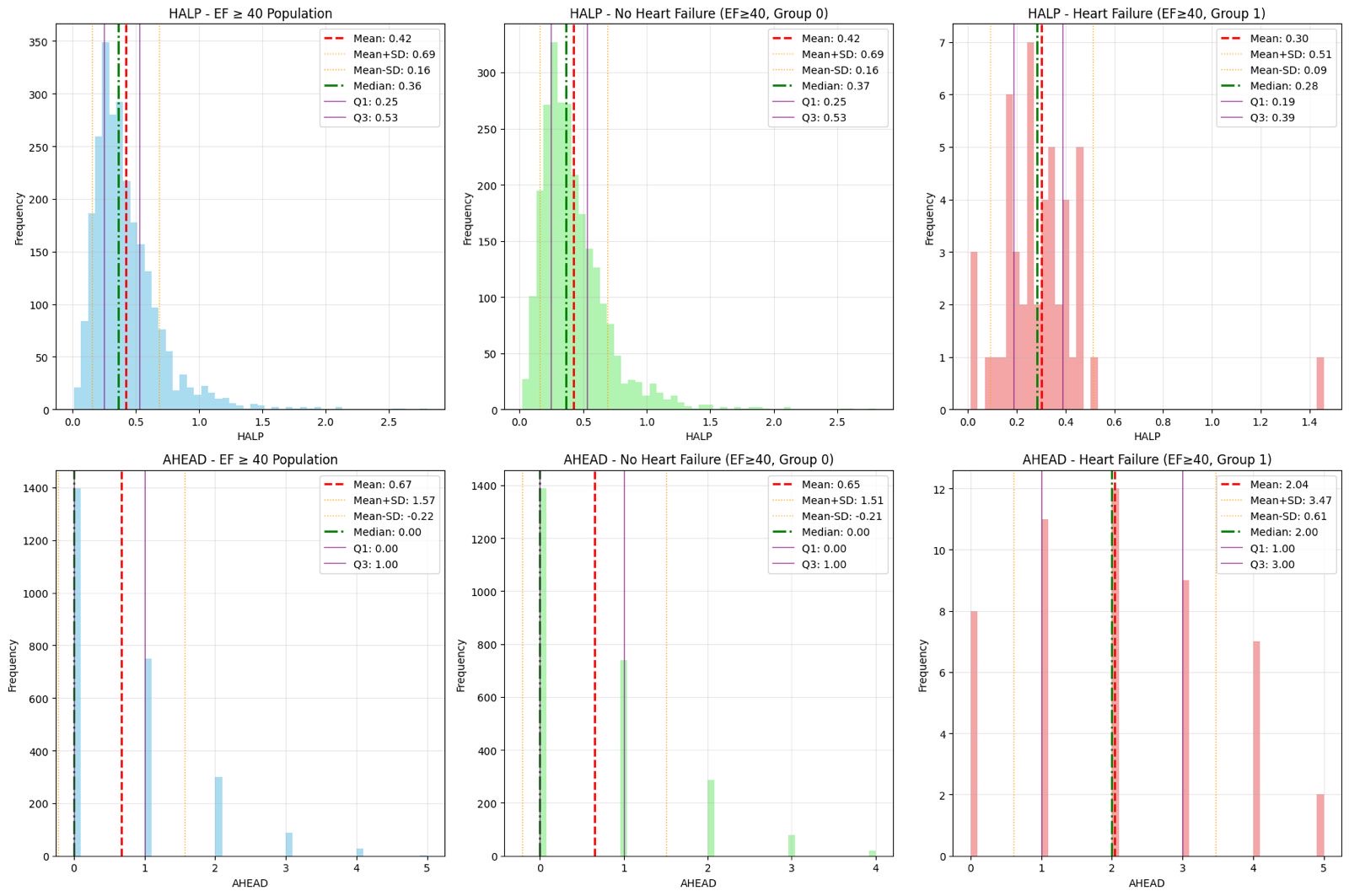

Supplement: Supplementary file 1 [file jcm-15-04363-s001.zip › Supplemental Figure 6.JPG]

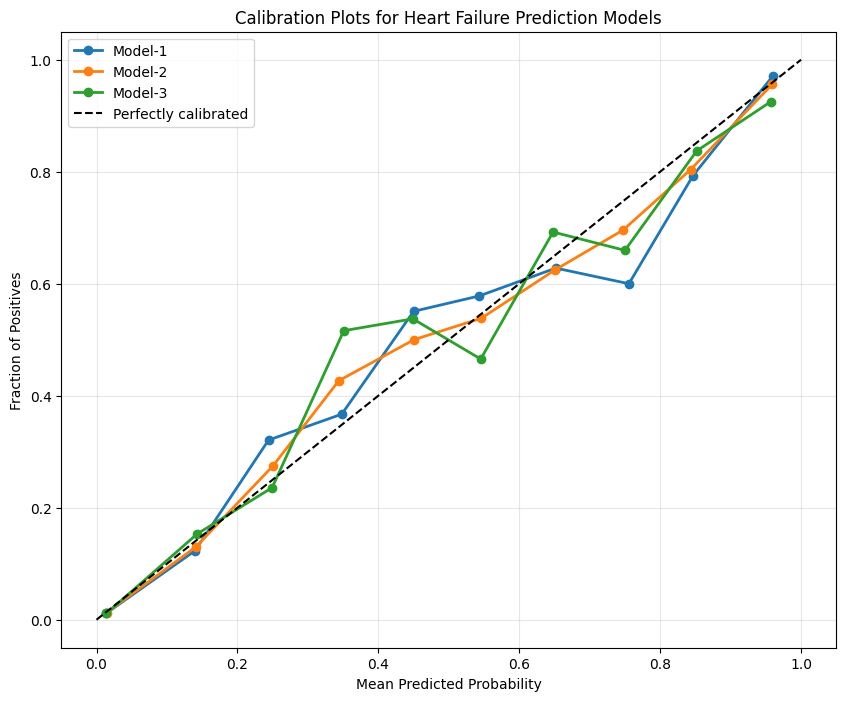

Supplement: Supplementary file 1 [file jcm-15-04363-s001.zip › Supplemental Figure 1.JPG]

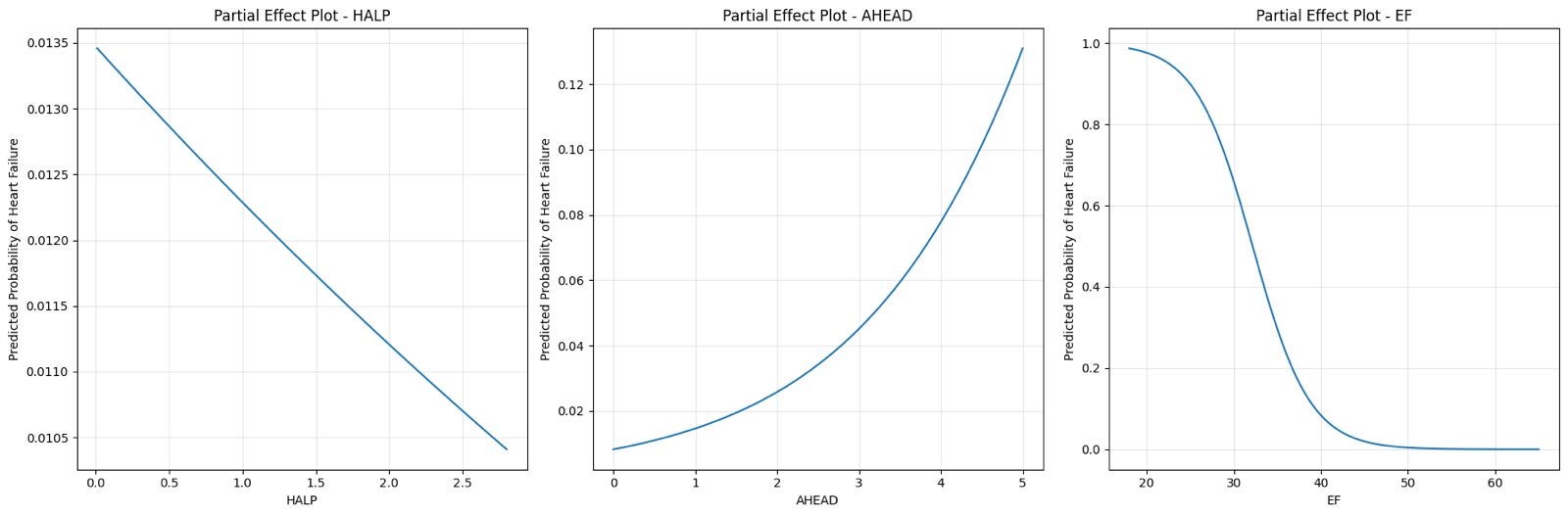

Supplement: Supplementary file 1 [file jcm-15-04363-s001.zip › Supplemental Figure 2.JPG]

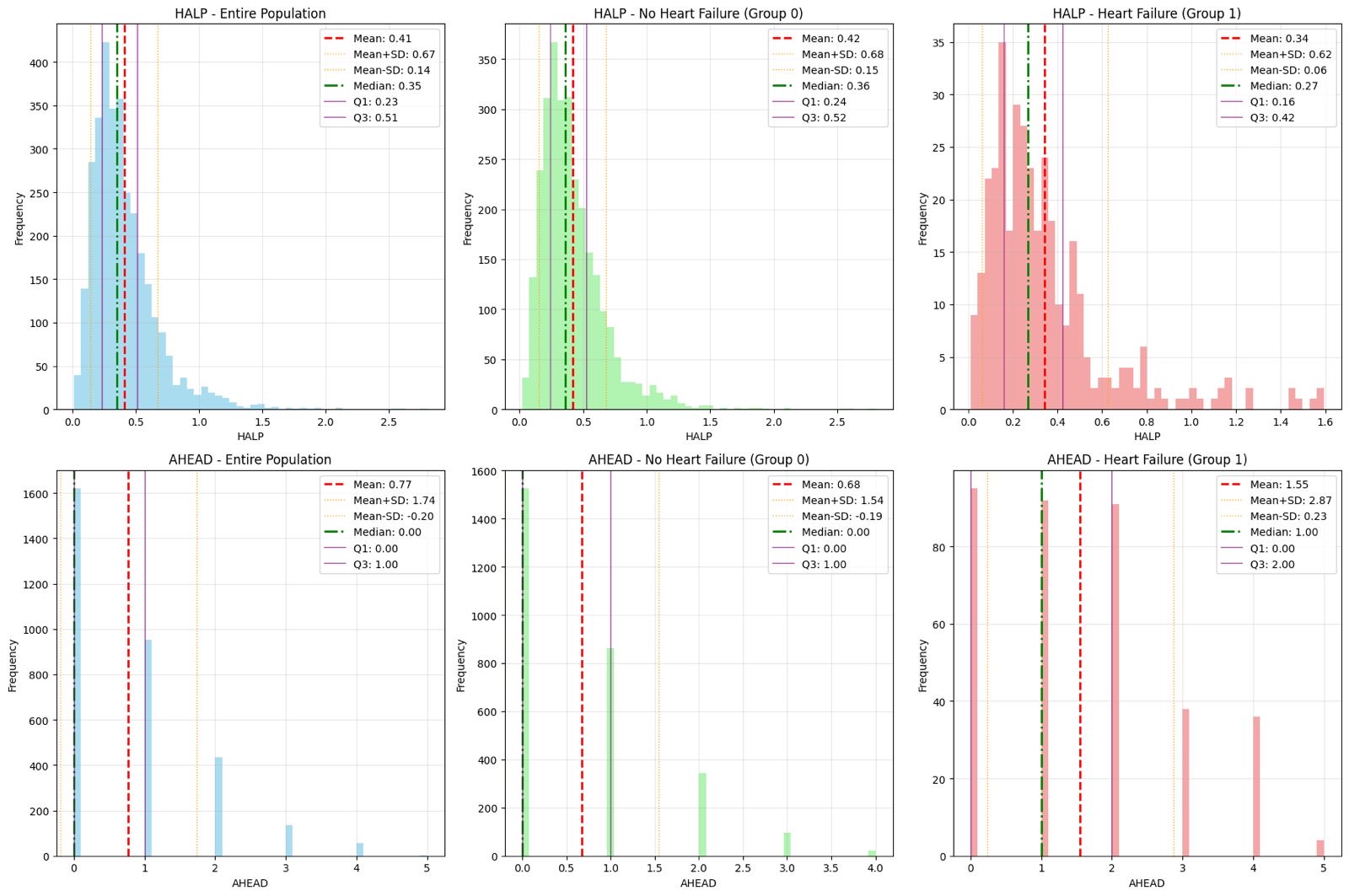

Supplement: Supplementary file 1 [file jcm-15-04363-s001.zip › Supplemental Figure 3.JPG]

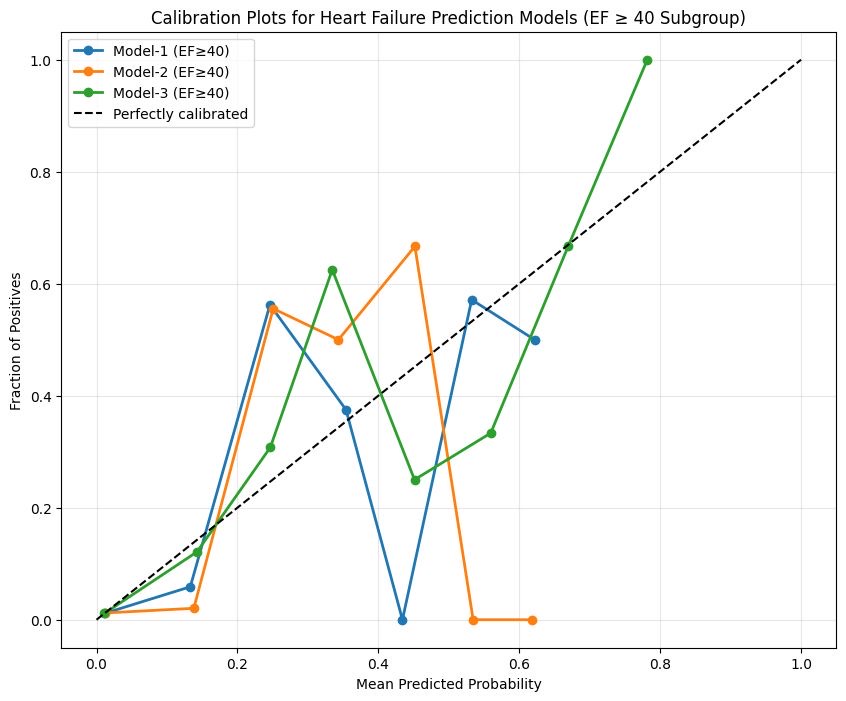

Supplement: Supplementary file 1 [file jcm-15-04363-s001.zip › Supplemental Figure 4.JPG]

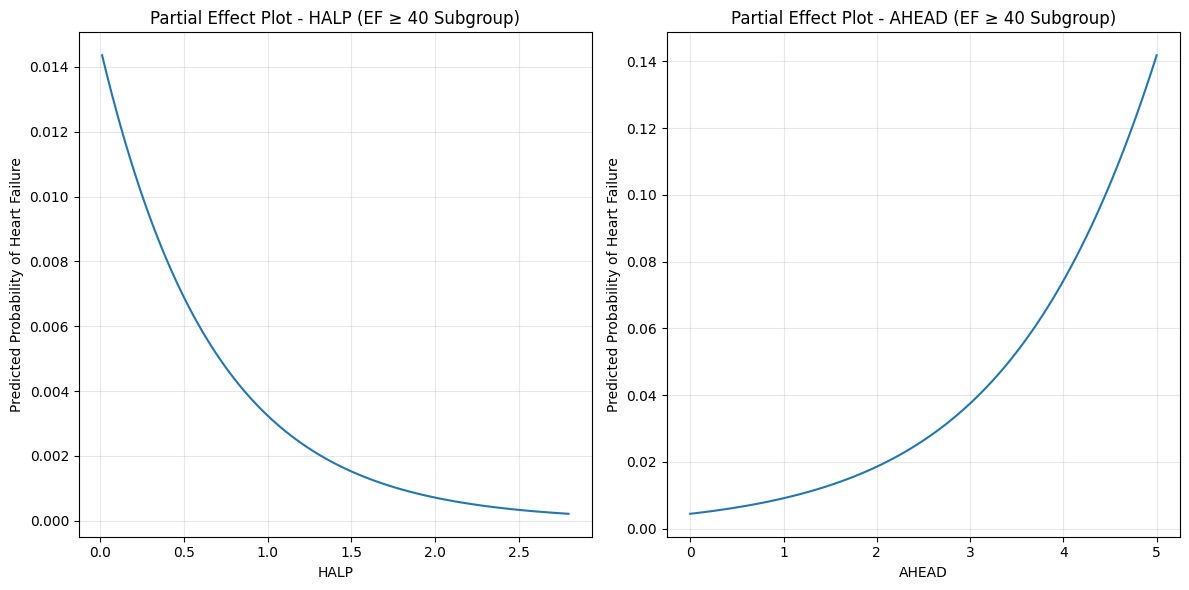

Supplement: Supplementary file 1 [file jcm-15-04363-s001.zip › Supplemental Figure 5.JPG]
